# Towards full clinical trial registration and results publication: longitudinal meta-research study in Northwestern and Central Switzerland

**DOI:** 10.1186/s12874-023-01840-9

**Published:** 2023-01-27

**Authors:** Katharina Klatte, Constantin Sluka, Viktoria Gloy, Ala Taji Heravi, Christof Schönenberger, Nienke Jones, Elena Brunnschweiler, Christiane Pauli-Magnus, Matthias Briel

**Affiliations:** 1 Department of Clinical Research, University Hospital Basel and University of Basel, Spitalstrasse 12, 4031 Basel, CH Switzerland; 2 Ethics Committee Northwest and Central Switzerland, Basel, Switzerland; 3 Department of Health Research Methods, Evidence, and Impact, McMaster University, Hamilton, Ontario Canada

**Keywords:** Randomized controlled trials, Trial registration, Legal obligation, Non-registration reasons, Switzerland

## Abstract

**Objective:**

The registration of clinical trials is required by law in Switzerland. We investigated (1) the proportion of registered and prospectively registered clinical trials, (2) the availability of results for ethically approved trial protocols, (3) factors associated with increased registration, and (4) reasons for non-registration.

**Design and setting:**

We included all clinical trials with mandatory prospective registration, which were approved by the ethics committee of Northwestern and Central Switzerland between January 1, 2016, and December 31, 2020.

**Methods:**

We extracted relevant trial characteristics from the Swiss Business Administration System for Ethics Committees and systematically searched the International Clinical Trials Registry Platform and primary trial registries for corresponding registry entries. We used multivariable logistic regression to examine the association between trial characteristics and registration. We qualitatively assessed reasons for non-registration of trials through an email questionnaire for trial investigators.

**Results:**

Of 473 included clinical trials, 432 (91%) were registered at all and 326 (69%) were prospectively registered. While the percentages of registration and prospective registration of investigator-sponsored trials increased from 85 to 93% and from 59 to 70% over 5 years, respectively, industry-sponsored trials consistently remained at a high level of prospective registration (92 to 100%). Trials with multiple centres, higher risk category, or methodological support from the local clinical trials unit were independently associated with increased registration rates. Of 103 clinical trials completed before August 2020, results were available for 70% of industry-sponsored trials and 45% of investigator-sponsored trials as peer-reviewed journal publications or in trial registries. Most common reasons for non-registration provided by investigators were lack of time or resources (53%), lack of knowledge (22%), and lack of reminders by the ethics committee (36%).

**Conclusions:**

In Northwestern and Central Switzerland about 10% of clinical trials remained unregistered despite the obligation by law. More support for investigators and stricter enforcement by regulators are needed to improve the transparency of investigator-sponsored trials in particular.

**Supplementary Information:**

The online version contains supplementary material available at 10.1186/s12874-023-01840-9.

## Background

Trial registries create a public record of all planned, ongoing, and completed clinical trials. Hereby, clinical trial registries help to detect unnecessary duplication of research and publication bias [1]. Through prospective documentation of important trial characteristics such as the primary outcome, eligibility criteria, or planned sample size trial registration further helps to minimize selective outcome reporting, ‘spin’, or other bad research practices [2–5]. Registration of all clinical trials as well as timely publication of trial results are important aspects addressing the need for transparency in clinical research [6] and constitute a big step towards “Open Science” [7–9]. In 2004, the International Committee of Medical Journal Editors (ICMJE) recommended publishing trial reports only if the trial was registered [10]. The World Medical Association included a statement in the Declaration of Helsinki that “every research study involving human subjects must be registered” [11]. Further, the Federal Drug Administration (FDA) expanded their “Final Rule” upon the requirement with additional data elements for both registration and results submission records in 2017 [12]. In Switzerland, prospective registration of a clinical trial in a primary trial registry such as clinicaltrials.gov or European Union Clinical Trials Register (EUCTR), for instance, has been made mandatory by law in 2014 (Art 56 Human Research Act) [13]. The International Clinical Trials Registry Platform (ICTRP) of the World Health Organization (WHO) provides an overview of all clinical trials registered in one of the considered primary trial registries. These primary registries meet specific criteria for content, quality and validity, accessibility, unique identification, technical capacity and administration as well as the requirements of the ICMJE [14, 15].

Various studies have already examined trial registration and, in particular, prospective trial registration based on published randomized controlled trials (RCTs) [3, 16–20]. A systematic review reported a pooled proportion of prospectively registered RCTs across 5529 RCTs of 20% [21]. In terms of improvement over time the meta-regression model reported in the review suggested that the proportion of prospectively registered trials across a wide range of clinical specialties increased from 3% in 2009 to 21% in 2013 (18% increase, *p* = 0.04) [21]. Another systematic review of clinical trials published in major respiratory journals between 2010 and 2018 found an increase for prospective trial registration rates - from 75% in 2010 up to 100% in 2018 [22]. However, the group of published trials does not comprise all trials approved by an ethics committee and, therefore, the generalizability of these findings may still be limited. An international meta-research study of 326 RCT protocols approved in 2012, which included 165 RCT protocols from Switzerland, found that one in five trials (70/326) remained unpublished at 10 years follow-up, and 21% of those unpublished trials (15/70) were not registered, i.e. they remain undetectable for the research community and the public [23]. Furthermore, an analysis of trials, required to register under the Food and Drug Administration Amendments Act (FDAAA) of 2007, by the “Trials Tracker” initiative revealed in 2020 that only 41% of trials from all sponsors have reported their results at clinicaltrials.gov 1 year after trial completion [24]. However, the sensitivity of such automated search processes for trial results has not been examined yet in a local context beyond specific registries such as clinicaltrials.gov [25–27] or EUCTR [28].

In view of these findings, further action is needed to increase compliance with registration and publication requirements to improve clinical research transparency and, hereby, promote public trust. Having a national law in place that mandates prospective trial registration is an important step, however, it needs to be implemented and enforced in local research environments to achieve its intended purpose. We, therefore, investigated in close collaboration with the local Ethics Committee of Northwestern and Central Switzerland (EKNZ) (1) the proportion of registered and prospectively registered clinical trials and (2) the availability of trial results for protocols approved between 2016 and 2020, (3) factors associated with trial registration rates including the use of methodological support provided by the Clinical Trials Unit (CTU) at the University Hospital Basel, (4) the sensitivity of automated publication tracking through the “Trials Tracker” approach in Northwestern and Central Switzerland, and (5) reasons for non-registration.

## Methods

### Study sample

Since January 1, 2016, it is mandatory to submit all study protocols for approval to a research ethics committee centrally via the Business Administration System for Ethics Committees (BASEC) in Switzerland. Through a research partnership with the EKNZ, we were granted access to the BASEC data exports under a confidentiality agreement though many fields from the database are publicly accessible through the Swiss National Clinical Trials Portal (https://www.kofam.ch/en/snctp-portal/searching-for-a-clinical-trial). In the present study, we included all studies that were (1) classified as clinical trials (ClinV, clinical intervention studies) in BASEC and (2) approved by the EKNZ between January 1, 2016, and December 31, 2020.

### Data collection

For all included trials we extracted relevant characteristics such as number of intervention arms, sponsorship, and target sample size from BASEC. Using a provided registry number, the trial title, patient population, intervention, or specific outcomes we systematically searched the ICTRP of the WHO for corresponding registry entries of all included trials. We used a cloud-based database for data collection (squiekero). Two trained researchers performed the registry search and data extraction for each included study independently and in duplicate. Disagreements were resolved by discussion and consensus. If a registry entry could not be found for a trial on ICTRP, we consecutively searched clinicaltrials.gov and EUCTR, and finally conducted a Google search. The searches were carried out between April 28, 2020 and April 21, 2021. For all trials for which we could not identify a registration entry through electronic searches, we surveyed corresponding trial investigators (documented in BASEC) for further information about trial registration. If investigators provided a registration number until Sept 1, 2021, corresponding trials were classified as registered in our data set. If contacted investigators did not provide a valid registration number for a trial, we eventually considered that trial not registered. From identified registry records we extracted further trial information such as date of registration, date of first patient enrolled, actual sample size, status of the trial, and sponsorship. We examined prospective registration (i.e. the number of trials registered prior to commencement of participant enrolment) as well as a more conservative measure of timely registration defined as registration within 30 days of trial commencement [3, 23]. All trials registered after enrolment of the first patient or after 30 days from enrolment of the first patient, respectively, were classified as retrospectively registered. To inquire about reasons for non-registration we sent a questionnaire to all principal investigators of trials not registered at the time of data extraction (*n* = 60) via email. In addition, the questionnaire aimed to assess investigators’ awareness of trial registration obligations, and to explore obstacles for trial registration (see supplementary material for full questionnaire). Responses of investigators providing a registration number in the questionnaire and considered registered in the qualitative analysis (*n* = 19) were still included in the analysis of quantitative outcomes (41 non-registered trials, 19 registered trials). Clinical trials making use of CTU services were identified by systematically searching internal CTU files containing meta-information of all CTU-supported studies and checking for BASEC ID numbers. In order to identify results publication of completed trials, we conducted a systematic search of the following three electronic databases for full-text publications corresponding to main research question stated in the registry: PubMed, Google Scholar, and Trial Registries. We used keywords of the trial title/objective and verified identified publications with trial outcomes, sample size and timeframe of trials stated in the trial registry. For all corresponding full text publications identified, we extracted type of publication, date of publication, and the registry number if provided. All searches and data extraction were conducted in duplicate, and disagreements were resolved by discussion.

### Data analysis

Quantitative data about trial registration, prospective trial registration, results publication, and reasons for non-registration were summarized as frequencies and percentages, stratified by sponsorship (industry- compared to investigator-sponsored). We conducted univariable and multivariable logistic regression analyses with trial registration as dependent variable and sponsorship (industry- compared to. investigator-sponsored), multicenter compared to single center trials, risk category of trial (low, medium, high), and use of CTU services (yes compared to no) as independent variables. We hypothesized that industry-sponsorship, multicenter trials, higher risk category, and use of CTU services, were associated with higher prevalence of trial registration and prospective trial registration. For all regression models, we calculated unadjusted and adjusted odds ratios (ORs) with 95% confidence intervals (CIs), and *p*-values. In the adjusted analyses we included all above mentioned independent variables in the regression model. We evaluated the sensitivity of automated processes as used with the “Trials Tracker” by comparing our findings on results publication with an automated process based on a registry such as Clinicaltrials.gov. All quantitative analyses were conducted using R version 3.5.3 (Ppackages: deplyr, tidyr, tidyverse, magrittr, lubridate, tables, scales, stringr, ggplot2; see supplementary material for analysis code). We qualitatively analysed open-ended questions about reasons for non-registration using content analysis [29].

## Results

### Study sample characteristics

Of 473 clinical trials approved by the EKNZ between 2016 and 2020, 342 (72.3%) were investigator-sponsored and 323 (68.3%) used a randomized design (Table 1). The median planned sample size for Switzerland was 32 participants (interquartile range [IQR] 16 to 75). Two hundred eighteen studies (46.1%) were multicentre of which most were international (78.9%; 172/218). Approximately half of the trials were classified as low risk according to the Swiss Human Research Act.

**Table 1:** Characteristics of included clinical trials

Characteristics	Categories	All trials (***n*** = 473)	Investigator-sponsored trials (***n*** = 342)	Industry-sponsored trials (***n*** = 131)
Target sample size in Switzerland (median, IQR)		32 (16-75)	45 (22-100)	15 (8-28)
Trial intervention, n (%)	Drugs	215 (45.5)	116 (33.9)	99 (75.6)
	Medical devices	96 (20.3)	69 (20.2)	27 (20.6)
	Behavioral	33 (7.0)	33 (9.6)	0 (0.0)
	Diagnostic	28 (5.9)	26 (7.6)	2 (1.5)
	Rehabilitation	23 (4.9)	23 (6.7)	0 (0.0)
	Dietary supplements	18 (3.8)	18 (5.3)	0 (0.0)
	Surgical	16 (3.4)	15 (4.4)	1 (0.8)
	Other^b^	44 (9.3)	42 (12.3)	2 (1.5)
Trial design, n (%)	Single arm	121 (25.6)	78 (22.8)	43 (32.8)
	Multiple arms^c^	352 (74.4)	264 (77.2)	88 (67.2)
	Randomized	323 (68.3)	239 (69.9)	84 (64.1)
	Non randomized	29 (6.1)	25 (7.3)	4 (3.1)
Risk category^a^, n (%)	Low risk	238 (50.3)	220 (64.3)	18 (13.7)
	Intermediate risk	83 (17.6)	71 (20.8)	12 (9.2)
	High risk	152 (32.1)	51 (14.9)	101 (77.1)
Trial sites, n (%)	Single Center	255 (53.9)	243 (71.1)	12 (9.2)
	Multicenter	218 (46.1)	99 (28.9)	119 (90.8)
	National	46 (9.7)	43 (12.6)	3 (2.3)
	International	172 (36.4)	56 (16.4)	116 (88.5)
Use of CTU service, n (%)		104 (22.0)	104 (30.4)	0 (0.0)

*Abbreviation*: *IQR* interquartile range 25% percentile - 75% percentile, *CTU* Clinical Trials Unit

^a^Classification of studies in the Human Research Act: Category A – low risk for trials with products authorized in Switzerland, and used according to Swiss Summary of Product Characteristics; Category B - intermediate risk for trials with products authorized in Switzerland, not used according to Swiss Summary of Product Characteristics; Category C - high risk for trials with products not authorized in Switzerland. Intermediate and high risk categories require additional authorization by federal authority (Swissmedic) [ 30]

^b^Includes: exercise trials, physiotherapy, transplant products, PK/PD safety trials, radiation therapy, palliation, other diet trials

^c^ Includes cross-over (*n* = 71), parallel group (*n* = 278), factorial (*n* = 3)

**Fig. 1:**
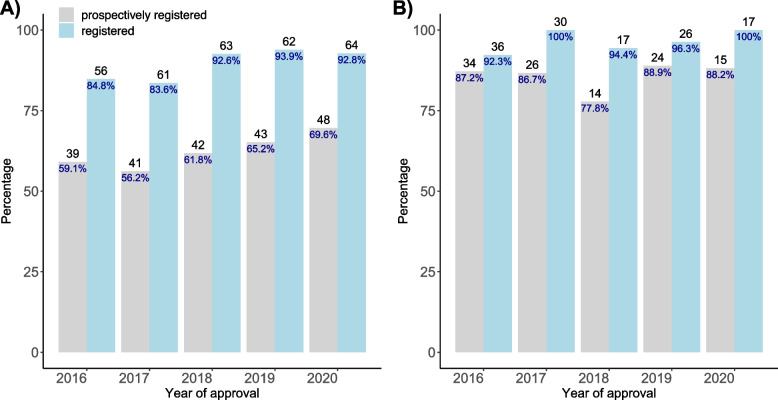
Percentage of clinical intervention studies registered and prospectively registered from 2016 to 2020 stratified by sponsorship. Panel A: Investigator-sponsored studies, Panel B: Industry-sponsored studies

Of all 473 clinical trials, 432 (91.3%) could be identified in a primary registry either via our sensitive search strategy or by contacting the investigators directly (Table 2). There were 326 trials registered prospectively (68.9%), and using a more conservative definition of prospective registration within 30 days of enrolling the first participant, there were 371 trials (78.4%) prospectively registered. Prospective registration was more prevalent in industry-sponsored trials than in investigator-sponsored trials (86.3% compared to. 62.3%, Table 2). Over the observation time of 5 years, there was a trend of increasing registration in investigator-sponsored trials with an increase in prospective trial registration from 59.1% in 2016 to 69.6% in 2020 (Fig. 1, Panel A), while industry-sponsored trials remained a high level of registration throughout (Fig. 1, Panel B; without stratification and with more conservative definition of prospective registration see Supplementary Fig. 1a-d).

**Table 2:** Registration status of EKNZ approved clinical trials 2016-2020

Registration status	All trials (***n*** = 473)	Investigator-sponsored trials (***n*** = 342)	Industry-sponsored trials (***n*** = 131)
**Registered (n, %)**	432 (91.3%)	306 (89.5%)	126 (96.2%)
**Prospectively**^a^	326 (68.9%)	213 (62.3%)^c^	113 (86.3%)
**Prospectively 30**^b^	371 (78.4%)^b^	249 (72.8%)^c^	122 (93.1%)

^a^ Before first patient enrolled

^b^ Before or within one month (− 30 days) of first patient enrolled

^c^ Five studies without date of first patient enrolled

### Trial characteristics associated with registration

We found that higher risk categories (intermediate and high), multicenter studies, and use of CTU services were independently associated with increased study registration (Table 3). We found similar results for prospective registration (Supplementary Table 1).

**Table 3:** Associations between trial characteristics and registration status in logistic regression

Characteristics^**a**^	Registered ***n*** = 432	Non- registered ***n*** = 41	Univariable			Multivariable^**b**^		
			OR	95% CI	***p***-value	OR	95% CI	***p***-value
Single center (compared to multicenter)	219 (85.9%)	36 (14.1%)	0.14	0.05-0.34	< 0.001	0.20	0.064-0.60	0.003
Investigator (compared to industry) sponsorship	306 (89.5%)	36 (10.5%)	0.34	0.11-0.81	0.026	1.66	0.64-7.59	0.42
Risk category low	201 (84.5%)	37 (15.5%)	Reference			Reference		
Risk category intermediate	81 (97.6%)	2 (2.4%)	7.45	2.21-46.52	0.006	5.26	1.74-37.54	0.026
Risk category high	150 (98.7%)	2 (1.3%)	13.81	4.14-85.74	< 0.001	9.00	2.56-71.32	0.008
Use of CTU service (compared to. no service)	103 (99.0%)	1 (1.0%)	12.52	2.67-223.52	0.013	15.63	3.24-281.23	0.007

*Abbreviations*: *OR* odds ratio, *CI* confidence, *CTU* Clinical Trials Unit

^a^Reference values: sample size < 100, multi-center trials, investigator-initiated trials and drug trials

^b^The variables Single center/ Multicenter, Sponsorship, Risk category, and Use of CTU services were included in the multivariable logistic regression

### Availability of trial results

Of 103 registered clinical trials with a completion date before August 2020, 58 (56.3%) had publicly available results until September 2021; in 51 trials (49.5%) results were published in a peer-reviewed journal, 16 (15.5%) trials provided results via a trial registry, and 7 trials did both. Of the 51 journal publications, 29 (56.9%) explicitly reported the registration number (Table 4). The percentage of reported trial results at 12 months after study completion was 69.2% for industry-sponsored trials, and 45.4% for investigator-sponsored trials; 53.8% of industry-sponsored trials reported results in a trial registry versus 2.6% of completed investigator-sponsored trials.

**Table 4:** Availability of results in completed clinical trials

Completed registered studies^**a**^(n)	All trials (***n*** = 103)	Investigator-sponsored trials (***n*** = 77)	Industry-sponsored trials (***n*** = 26)
**Publicly available trial results, 12 month after study completion**	58 (56.3%)	40 (45.4%)	18 (69.2%)
**Publication of results in registry**	16 (15.5%)	2 (2.6%)	14 (53.8%)
**Publication in peer-reviewed journal**	51 (49.5%)	38 (49.4%)	13 (50%)
Journal publication mentioned Registration Number^b^	29 (56.9%)	18 (47.4%)	11 (84.6%)

^a^Completed by August 2020 according to status of study provided in the registry

^b^Percentage of journal publications

With respect to the sensitivity of an automated approach searching for trial publications such as the “Trials Tracker”, we noted that an automated approach searching primary registries does not consider non-registered studies; in our sample, 41 of 473 studies (8.7%) approved by the EKNZ could not be identified in any primary registry. If the automated approach considers clinicaltrials.gov only (Trials Tracker), studies registered in other primary registries are missed. In our sample, 72.9% (345/432) of registered studies were registered in clinicaltrials.gov, and 18.4% (87/432) were exclusively registered in another primary registry (Supplementary Fig. 2). Thus, 27.1% (128/473) of studies would be missed through an automated export from clinicaltrials.gov. Considering additionally that only 56.9% of identified results publications explicitly mentioned the registration number, automated searching for the study registration number via PubMed likely misses a substantial number of study publications.

#### Reasons for non-registration and investigators’ awareness of registration facts

In total, 36 out of 60 contacted investigators returned a filled questionnaire (60% response rate). 19 of the corresponding trials were eventually identified as registered through the questionnaire, while 41 remained in the non-registered group. Overall, 27 (75.0%) of contacted investigators were aware of the obligation to register a clinical trial, and 14 (38.9%) were aware that the Swiss National Trials Portal (SNCTP-KOFAM) is not a primary registry (Table 5). Most researchers stated to know one of the common primary registries. Of the suggested barriers in the registration process listed in the questionnaire, the most commonly stated barrier was lack of “Time and Resources” (50.0%), followed by “Missing reminder of obligation to register the study” (36.1%). Most respondents did not take advantage of any CTU or CRO support services. Individual reasons for non-registration included researchers’ view that their study was not a clinical trial and un-awareness of the obligation among others.

**Table 5:** Survey of trial investigators with non-registered studies as of April 2020

Topic	36 of 60 investigators filled out the questionnaire, 24 did not respond			
**General awareness of Researchers**	Prospective registration is required by law	Registration is required before first participant enters the study	Swiss National Clinical Trials Portal (SNCTP)^a^ is not a primary registry	Registration is reasonable
	27 (75.0%)	26 (72.2%)	14 (38.9%)	32 (88.9%)
**Study support by service team**	Clinical Trials Unit	Contract Research Organisation	Others	No support service
	7 (19.4%)	3 (8.3%)	2 (5.6%)	24 (66.7%)
**Knowledge of primary registries**	ClinicalTrials.gov	German Clinical Trials Register (DRKS)	EU Clinical Trials Register (EUCTR)	ISRCTN-Register
	32 (88.9%)	13 (36.1%)	14 (38.9%)	7 (19.4%)
**Perceived Barriers to study registration**	Insufficient knowledge of primary registries/ registration processes:	Limited time/ resources for registration process	Missing reminder of obligation to register the study	Others^b^
	8 (22.2%)	18 (50.0%)	13 (36.1%)	6 (16.7%)
**Stated reasons for non-registration**	- Study postponed/ unclear study start date (*n* = 2) - Missing local SOPs for registration (*n* = 1) - Unclear interpretation of regulations for Phase I studies (*n* = 1) - Study not considered as clinical trial by investigator (*n* = 4) - Unaware of the obligation to register (*n* = 2) - Short study, retrospective registration considered as unnecessary/confusing (*n* = 1) - One researcher responsible for all registrations in the research institute (*n* = 1) - No reason specified (*n* = 24)			

*Abbreviations*: *ISRCTN* International Standard Randomized Controlled Trial Number, *SOP* Standard Operating Procedure

^a^In Switzerland every study approved by an ethics committee and registered in a primary registry will be listed on the Swiss National Clinical Trials Portal (SNCTP)

^b^Others included unclear definition of the study, unclear responsibilities for registration within institution, COVID-19 induced delay

## Discussion

Our empirical study of 473 clinical trials with mandatory registration found that registration and prospective registration increased for investigator-sponsored trials over time but still needing further improvement, while industry-sponsored trials had high registration levels throughout the 5 years of observation. Multicenter studies and studies in a higher risk category were associated with increased registration, probably reflecting more intense supervision / control of those studies. In addition, 99% of investigator-sponsored trials with CTU support were registered suggesting an effective process at the CTU to ensure trial registration. Overall, results were made available for 70% of completed industry-sponsored trials and 45% of investigator-sponsored trials. Only about 3% of completed investigator-sponsored trials had results published in a registry, whereas 54% of industry-sponsored trial results were available in registries. Automated tracking of results publications of approved clinical trials proved challenging in our regional context due to a considerable proportion of unregistered trials, an appreciable distribution of trials registered in a number of different registries, and insufficient reporting of the registration number in trial publications. Reasons for non-registration provided by investigators included lack of time/resources, lack of knowledge, and lack of enforcement by ethics committees.

### Strengths and limitations

The strengths of this study include a comprehensive sample of all clinical trials approved between 2016 and end of 2020 in the jurisdiction of the EKNZ and full access to all study information in BASEC. We conducted a sensitive search for registry entries supplemented by a survey of investigators. We limited the number of variables in our regression models to reduce the probability of spurious associations. Finally, we complemented our quantitative analyses by a qualitative investigation of registration barriers.

Our study has the following limitations: First, our sample size was modest limiting the precision of stratified analyses over time. In some categories, for example industry-sponsored single center trials or industry-sponsored low risk trials, the sample size was very low. Second, only 36 of 60 contacted investigators of non-registered trials returned a filled questionnaire compromising our qualitative analysis and leaving the completion status for 24 trials unclear. Researchers responding to the survey may have a more positive view towards trial registration. Third, our sample was limited to trials approved by one Swiss ethics committee; therefore, our findings cannot be automatically extrapolated to other Swiss ethics committees or other countries. Fourth, in our analysis of results publication we only verified if the primary research question submitted to the ethics committee was addressed, but did not specifically check for outcome switching by comparing outcome information in trial registries and reported results.

### Comparison with other studies

A recently published meta-research study found that 6% of RCTs from a sample of 326 RCT protocols approved in 2012 by research ethics committees in Switzerland, UK, Germany, and Canada were not registered, with non-registration being more common among non-published RCTs [23]. The proportion of prospectively registered RCTs was 84%, which is slightly higher than the proportion in our study sample (78.4%). In our sample around 9% of trials were not registered. A systematic review and meta-analysis published in 2018 found that in different medical specialties, 2-79% of RCTs were not registered [21], which shows a large variation depending on medical specialty. In addition, proportions of study registration may dependent also on the study sample (published, approved) and the countries involved. A recently published editorial by DeVito and Goldacre summarized the current trial reporting in the EU [31]; while progress has been observed in terms of trial results published [28], it is mainly driven by a few countries [32, 33]. The different timeframes of the assessment also provide an explanation for the wide range of proportions found in different studies [21, 23, 34, 35].

In agreement with our results a systematic review on clinical registration in major respiratory journals reported that single center studies were more likely to be retrospectively registered or not registered [22]. An analysis of clinical trials approved in Switzerland from 2016 to 2020 showed that more than half of the trials were monocentric trials [36]. Since awareness and regulatory control might also be less in monocentric trials, education and support of the registration and dissemination processes for all research facilities in Switzerland should be aspired. In a survey of 149 researchers who had retrospectively registered a trial on ANZCTR between 2010 and 2015, the majority (56%) of survey respondents cited lack of awareness as a reason for not registering their study prospectively [37]. Seventy-four per cent stated that linking registration to ethics approval would facilitate prospective registration. A survey conducted by Mayo-Wilson et al. in the United States before the “The Final Rule” made trial registration and results reporting more tightly regulated, revealed that only a minority of academic organizations had policies and resources that facilitate clinical trial registration and reporting. They strongly suggested allocating resources to trial registration and reporting [38]. The medical university of South Carolina identified issues affecting their own compliance rate with FDAAA801 and evaluated newly implemented processes such as hiring a designated full time trial registration and reporting coordinator and a workflow that identifies trials early in the approval process requiring registration. Evaluation after 12 months demonstrated a marked increase to 98% overall compliance with the US federal regulations [39]. Similarly the Duke University and Johns Hopkins University implemented and evaluated supporting approaches which included training of investigators, implementation of an institutional policy, creation of centralized resources, intensive resourcing for results reporting, mandatory inclusion of registry numbers on billing claims, and implementation of proactive compliance measures [40, 41]. These implemented measures improved compliance at both health care institutions with a reduction of non-compliant trials from 44 to 2% over a 5 year period at the Johns Hopkins University, for instance [40]. These supporting initiatives are in agreement with our finding that the proportion of registration in trials with CTU service was 99%.

Besides the general obligation to register clinical trials and update registry information, a reliable linkage of publications to the registration number would increase the accuracy of automated processes that continuously provide information on trial result publication. Huser et al. also evaluated automated checking of trial registration ID in publications of five ICMJE founding journals, which revealed a registration in 88% of cases [42]. We only found a registration ID in 57% of trials published in journals. This difference is most likely explained by the sample of journals enforcing stricter rules for registration and including registration IDs. However, only looking at publication and their linkage to a registration number is not sufficient to identify trials where results are not available. Considering this limitation, the “Trials Tracker” initiative is now focusing on trials, which are required to report results on ClinicalTrials.gov or EUCTR and thus allow conclusive results to compliance of reporting on these platforms. In order to provide the complete content of research results of the scientific community, publication of results within the registries would reduce the likelihood of publication bias and spin [43, 44].

### Implications and future directions

Our study revealed encouraging results in terms of the development of registration rates over the last years, but further efforts are still needed. DeVito and Goldacre proposed that academic institutions should educate researchers about their responsibilities in terms of reporting and also ethics committees and funders should consider their responsibilities [31]. From our qualitative evaluation, a strong need for support in the registration process was identified and suggests that missing resources available for trial registration are often the reason for retrospective or non-registration. Education of investigators and support in the registration and publication processes would constitute important steps to more complete transparency of medical research. CTUs could catalyze these steps. Ethics committees may send email reminders to trial investigators informing them about their legal obligations and prospective trial registration should be stricter enforced by publishing journals [45].

During the Covid-19 pandemic, the importance of clinical research suddenly became publicly visible stressing the need for research transparency and availability of results [46]. Making study registration mandatory by law is an important step, which needs to be further enforced, but non-publication of trial results clearly remains insufficiently addressed, legally and academically [47]. In order to promote the patients’ and the public’s trust in clinical research, a legal obligation to publish clinical trial results in the format of a peer-reviewed article, preprint, or in a trial registry would be helpful. Until prospective trial registration and results publication are fully established, regular monitoring of both processes through meta-research is necessary, so that barriers can be timely identified and tackled. Further development of automated results publication tracking will be instrumental to scale up this task.

## Conclusions

Rates of registration and prospective registration have increased in investigator-sponsored trials over the past years in Northwestern and Central Switzerland, but still need further improvement. Multicenter trials and trials in a higher risk category were independently associated with increased registration. Almost all investigator-sponsored trials with CTU support were registered. Reported reasons for non-registration were lack of time/resources, lack of knowledge, and lack of enforcement by ethics committees. Availability of trial results was modest, particularly with respect to results publication of investigator-sponsored trials in registries. Automated tracking of results publications have to consider local settings in order to achieve sufficient sensitivity.

## Supplementary Information

**Additional file 1: Supplementary Figure 1a.** Prospective Registration (strict definition) 2016-2020 all studies. **Supplementary Figure 1b.** Prospective Registration (with 30 days of enrolment of first participant definition) 2016-2020 all studies. **Supplementary Figure 1c and 1d.** Percentage of clinical intervention studies registered and prospectively registered (with 30 days of enrolment of first participant definition) from 2016 to 2020 stratified by sponsorship. Panel C: Investigator-sponsored studies, Panel D: Industry-sponsored studies. **Supplementary Table 1.** Associations between trial characteristics and prospective trial registration. **Supplementary Table 2.** Association between the use of DKF Services and registration status. **Supplementary Figure 2.** Distribution of used trial registries for trials approved by the EKNZ. **Supplementary material:** Questionnaire. **Supplementary material:** Analysis code.

## Data Availability

The data that support the findings of this study are available from the Ethics Committee of Northwestern and Central Switzerland but restrictions apply to the availability of these data, which were used under license for the current study, and so are not publicly available. Data are, however, available from the authors upon reasonable request and with permission of the Ethics Committee of Northwestern and Central Switzerland.
