# Supplementary material for: Towards full clinical trial registration and results publication: longitudinal meta-research study in Northwestern and Central Switzerland
Source: BMC Med Res Methodol. 2023 Jan 27;23:27. doi: 10.1186/s12874-023-01840-9 (PMC9880919; doi:10.1186/s12874-023-01840-9)
Supplement: Supplementary file 1 — Additional file 1: Supplementary Figure 1a. Prospective Registration (strict definition) 2016-2020 all studies. Supplementary Figure 1b. Prospective Registration (with 30 days of enrolment of first participant definition) 2016-2020 all studies. Supplementary Figure 1c and 1d. Percentage of clinical intervention studies registered and prospectively registered (with 30 days of enrolment of first participant definition) from 2016 to 2020 stratified by sponsorship. Panel C: Investigator-sponsored studies, Panel D: Industry-sponsored studies. Supplementary Table 1. Associations between trial characteristics and prospective trial registration. Supplementary Table 2. Association between the use of DKF Services and registration status. Supplementary Figure 2. Distribution of used trial registries for trials approved by the EKNZ. Supplementary material: Questionnaire. Supplementary material: Analysis code. [file 12874_2023_1840_MOESM1_ESM.docx]

# Supplementary Material

**Supplementary Figure 1a: Prospective Registration (strict definition) 2016-2020 all studies**

**Supplementary Figure 1b: Prospective Registration (with 30 days of enrolment of first participant definition) 2016-2020 all studies**

**Supplementary Figure 1c and 1d: Percentage of clinical intervention studies registered and prospectively registered (with 30 days of enrolment of first participant definition) from 2016 to 2020 stratified by sponsorship. Panel C: Investigator-sponsored studies, Panel D: Industry-sponsored studies.**

**Supplementary Table 1: Associations between trial characteristics and prospective trial registration**

| **Trial characteristics*** | **Prospectively registered trials n= 326** | **Not prospectively registered trials n=142** | **Univariable** | | | **Multivariable** | | |
| --- | --- | --- | --- | --- | --- | --- | --- | --- |
|  |  |  | **OR** | **95% CI** | **p-value** | **OR** | **95% CI** | ***p*-value** |
| Single centre (vs. multicentre) | 141 (55.9%) | 111 (44.0%) | 0.21 | 0.13-0.33 | <0.001 | 0.32 | 0.18-0.56 | <0.001 |
| Investigator (vs. Industry) sponsorship | 213 (63.2%) | 124 (36.8 %) | 0.27 | 1.15-0.46 | <0.001 | 0.91 | 0.42-1.96 | 0.82 |
| Risk category low | 124 (52.8%) | 111(47.23%) | Reference | | | Reference | | |
| Risk category medium | 67 (81.7%) | 15 (18.3%) | 4.00 | 2.21-7.64 | <0.001 | 3.16 | 1.71-6.16 | <0.001 |
| Risk category high | 135 (89.4%) | 16 (10.6%) | 7.55 | 4.35-13.90.50 | <0.001 | 4.48 | 2.27-9.28 | <0.001 |
| CTU service | 80 (76.9%) | 24 (23.1%) | 2.08 | 1.14-4.06 | 0.023 | 2.36 | 1.35-4.23 | 0.0029 |
| *Reference values: sample size <100, multicentre trials, investigator-initiated trials and drug trials. | | | | | | | | |

Abbreviations: OR, odds ratio; CI, confidence; CTU, Clinical Trials Unit.

### Supplementary Table 2: Association between the use of DKF Services and registration status

| **Registration status** | **Investigator-sponsored trials with CTU Services (n=104)*** | **Investigator-sponsored trials without CTU Services (n=238)** | **All investigator-sponsored trials (n=342)** |
| --- | --- | --- | --- |
| **Registered, n (%)** | 103 (99.0 %) | 203 (85.3 %) | 306 (89.5 %) |
| **Prospectively registered, n (%)** | 80 (76.9 %) | 135 (56.7 %) | 215 (62.8 %) |

*104 (30.4 %) of investigator-sponsored trials made use of CTU services

Abbreviations: CTU, Clinical Trials Unit.

**Supplementary Figure 2: Distribution of used trial registries for trials approved by the EKNZ**


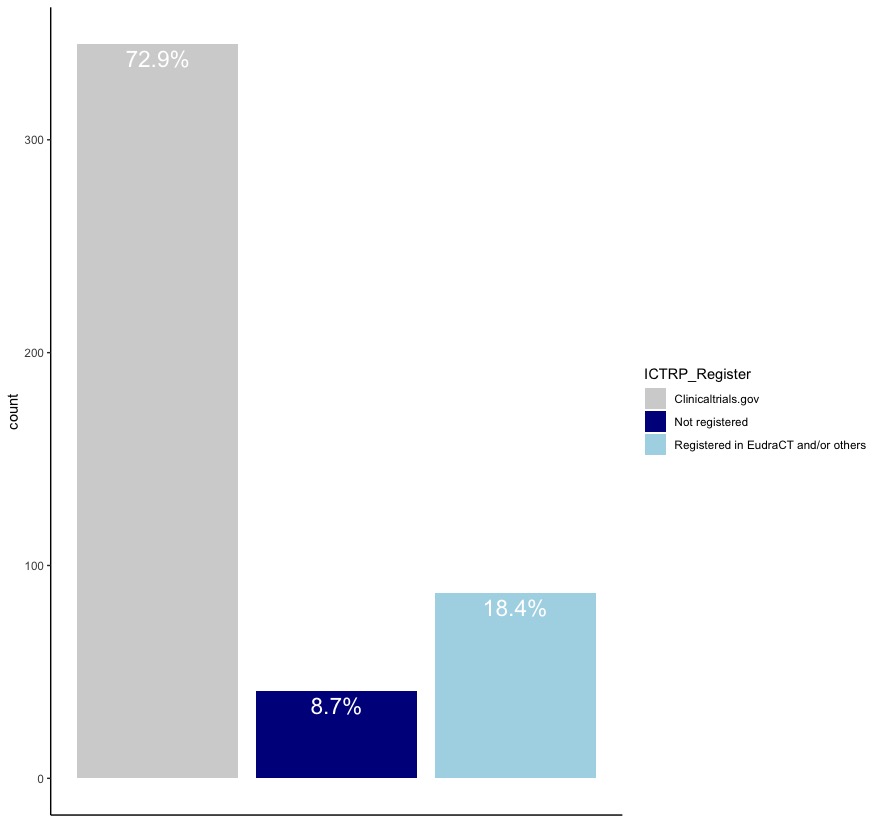


Abbreviations: ICTRP, International Clinical Trials Registry Platform; EudraCT, EU Clinical Trial Register.

**Supplementary material: Questionnaire**

**Applicant:**

**Title of Project:**

**Trial Registration**

1. Have patients already been enrolled since the approval of the study by the ethics committee?

**Yes** **No**

- - If **No**, (← for international studies):
    - Has the study not started yet
    - Has the study not started in Switzerland
  - If **No**, when is the start of the study planned in Switzerland? ___________________

1. Was the study registered in one of the primary registries?

**Yes** **No**

- - If **Yes**, please provide registration number­­­­­­­­­­­­: ___________________
  - **If No**, would you like the CTU to register the study **Yes
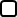
** **No
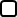
**

1. Are you aware that it is mandatory to register prospective clinical trials?

**Yes** **No**

1. Are you aware that clinical trials have to be registered before the first patient is enrolled?

**Yes** **No**

1. Are you aware of the primary registries of the World Health Organisation?
   - - ClinicalTrials.gov
     - German Clinical Trials Register (DRKS)
     - EU Clinical Trial Register (EU-CTR/EudraCT)
     - ISRCTN-Register (International Standard

Registered Clinical/soCial sTudy Number)

- - - Others __________________
    - Not aware of any of the Primary registries

1. Are you aware that the Swiss National Trials Portal (SNCTP- kofam) is not a primary registry?

**Yes** **No**

1. Did you take atvantage of a support service for the study?
   - - Clinical Trial Unit (CTU)
     - Contract Research Organisation (CRO)
     - Other: ___________________________
     - No support service
2. What barriers of trial registration do you perceive?

- Insufficient knowledge of primary registries
- Process of registration is unclear or unknown
- Limited time/ resources for registration process
- Missing support in the registration process
- Missing reminder of obligation to register the study
- Other

____________________________________

____________________________________

1. Do you consider Trial Registration as reasonable?

**Yes** **No**

Comments: _________________________________________________________________________________________________________________________________________________________________________________________________________________________________________________________________________________________________________________________________________

**Supplementary material: Analysis code**

**Code for Analysis is R**

**## TIMELINESS of registration**

###Prospectively registered studies

#### In clinicaltrials.gov ICTRP

df2<-RegisteredStudies %>%

filter(!(is.na(dateofregistryclinicaltrialt.govonICTRP)))%>%

filter(!(is.na(ICTRPclinicaltrial.govdatefirstpat)))

##Converting string to R date form

df2$dateofregistryclinicaltrialt.govonICTRP <- as.Date(df2$dateofregistryclinicaltrialt.govonICTRP, format = "%d.%m.%Y")

df2$ICTRPclinicaltrial.govdatefirstpat <- as.Date(df2$ICTRPclinicaltrial.govdatefirstpat, format = "%d.%m.%Y")

df2$diff_in_days<- difftime(df2$ICTRPclinicaltrial.govdatefirstpat,df2$dateofregistryclinicaltrialt.govonICTRP , units = c("days"))

## categorizing studies into Retro and Prospectively

df2 <- df2 %>%

mutate(timeliregist = ifelse (df2$diff_in_days >0, "Pro", "Retro"))

Prospectivelyregistered_Clingov <- df2%>%

select (id, timeliregist, ICTRP.gov.Sponsor,diff_in_days)

####In EudraCT

df3<-RegisteredStudies %>%

filter (ICTRP_short != "Clinicaltrials.gov")

df3<- df3%>%

filter(!(is.na(ICTRPeudraCTcountryspecdateofreg)))

df3$ICTRPeudraCTcountryspecdateofreg <- as.Date(df3$ICTRPeudraCTcountryspecdateofreg, format = "%d.%m.%Y")

df3$ICTRPeudraCTcountryspecdatefirstpat <- as.Date(df3$ICTRPeudraCTcountryspecdatefirstpat, format = "%d.%m.%Y")

df3$diff_in_days<- difftime(df3$ICTRPeudraCTcountryspecdatefirstpat,df3$ICTRPeudraCTcountryspecdateofreg , units = c("days"))

df3 <- df3 %>%

mutate(timeliregist = ifelse (df3$diff_in_days >0, "Pro", "Retro"))

Prospectivelyregistered_EudraCT <- df3%>%

select (id, timeliregist, EudraCT.Sponsor.of.the.Study, diff_in_days)

#### in other register

df4<-RegisteredStudies %>%

filter (ICTRP_short != "Clinicaltrials.gov")

df4<- df4%>%

filter(is.na(ICTRPeudraCTcountryspecdateofreg))%>%

filter(!(is.na(ICTRPotherregistrydateofregistration)))

df4$ICTRPotherregistrydateofregistration <- as.Date(df4$ICTRPotherregistrydateofregistration, format = "%d.%m.%Y")

df4$ICTRPotherregistrydatefirstpat <- as.Date(df4$ICTRPotherregistrydatefirstpat, format = "%d.%m.%Y")

df4$diff_in_days<- difftime(df4$ICTRPotherregistrydatefirstpat,df4$ICTRPotherregistrydateofregistration , units = c("days"))

df4 <- df4 %>%

mutate(timeliregist = ifelse (df4$diff_in_days >0, "Pro", "Retro"))

Prospectivelyregistered_Other <- df4%>%

select (id, timeliregist, Other.Registry.Sponsor.of.Study, diff_in_days)

##Logistic Regression

###Univariablee Models

Cd_registered_DKFall <-Cd_registered_DKFall%>%

select (Project_ID, DKFdossier)

df_full_cd <- merge (df_full, Cd_registered_DKFall , by= "Project_ID")

df_full_withoutcd <- df_full%>%

filter (! (Project_ID %in% df_full_cd$Project_ID ))

df_full_2<- bind_rows(df_full_cd,df_full_withoutcd )%>%

mutate (DKF_service = ifelse((is.na (DKFdossier)), "no service" ,"service"))

#risk category - categorical

model <- glm(as.factor(df_full$registered) ~

as.factor(risk_category),#2 levels (3 categories)

family=binomial(link='logit'),data=df_full,)

summary(model)

exp(cbind(OR = coef(model), confint(model))) # confidence intervals and OR

#sponsor

model1 <- glm(as.factor(df_full$registered) ~

as.factor(Sponsor2), #1 level (2 categories)

family=binomial(link='logit'),data=df_full,)

summary(model1)

exp(cbind(OR = coef(model1), confint(model1)))# confidence intervals and OR

#multicenter

model2 <- glm(as.factor(df_full$registered) ~

relevel(as.factor(multicenter_singlecenter), ref=1), #1 level

family=binomial(link='logit'),data=df_full,)

summary(model2)

exp(cbind(OR = coef(model2), confint(model2)))# confidence intervals and OR

#DKF service

modelDKF <- glm(as.factor(df_full_2$registration) ~

relevel(as.factor(DKF_service), ref=1), #1 level

family=binomial(link='logit'),data=df_full_2,)

summary(modelDKF)

exp(cbind(OR = coef(modelDKF), confint(modelDKF)))# confidence intervals and OR

## MultivariableRegise Model with all above variables

model5 <- glm(as.factor(df_full_2$registration) ~

relevel(as.factor(multicenter_singlecenter), ref=1)+ #1 level+

as.factor(Sponsor2)+ #1

as.factor((DKF_service)),

as.factor(risk_category),#2 levels (3 categories)

family=binomial(link='logit'),data=df_full_2,)

summary(model5)

exp(cbind(OR = coef(model5), confint(model5)))# confidence intervals and OR

model5 <- glm(as.factor(`registration`) ~

as.factor(Sponsor2)+#2

relevel(as.factor(multicenter_singlecenter), ref=1)+#1, change of reference category,

as.factor(DKF_service)+ #1

relevel(as.factor(risk_category), ref=1),#1, #change of reference category,

family=binomial(link='logit'),data=df_full_2,)

summary(model5)

exp(cbind(OR = coef(model5), confint(model5)))# confidence intervals and OR
